# Reconciling Mediating and Slaving Roles of Water in Protein Conformational Dynamics

**DOI:** 10.1371/journal.pone.0060553

**Published:** 2013-04-11

**Authors:** Li Zhao, Wenzhao Li, Pu Tian

**Affiliations:** 1 College of Life Science, Jilin University, Changchun, China; 2 College of Life Science and MOE Key Laboratory of Molecular Enzymology and Engineering, Jilin University, Changchun, China; Oak Ridge National Laboratory, United States of America

## Abstract

Proteins accomplish their physiological functions with remarkably organized dynamic transitions among a hierarchical network of conformational substates. Despite the essential contribution of water molecules in shaping functionally important protein dynamics, their exact role is still controversial. Water molecules were reported either as mediators that facilitate or as masters that slave protein dynamics. Since dynamic behaviour of a given protein is ultimately determined by the underlying energy landscape, we systematically analysed protein self energies and protein-water interaction energies obtained from extensive molecular dynamics simulation trajectories of barstar. We found that protein-water interaction energy plays the dominant role when compared with protein self energy, and these two energy terms on average have negative correlation that increases with increasingly longer time scales ranging from 10 femtoseconds to 100 nanoseconds. Water molecules effectively roughen potential energy surface of proteins in the majority part of observed conformational space and smooth in the remaining part. These findings support a scenario wherein water on average slave protein conformational dynamics but facilitate a fraction of transitions among different conformational substates, and reconcile the controversy on the facilitating and slaving roles of water molecules in protein conformational dynamics.

## Introduction

Protein dynamics is critical for their functions [1]–[4] and evolvability [5], and is to a great extent determined by the roughness of their potential energy surface (PES). Solvents play an indispensable role in shaping dynamic behaviour of proteins through molecular interactions that contribute to protein PES. Energy transferred from the first hydration shell to surface residues of cyclophilin A was computationally demonstrated to influence catalysis through network fluctuations [2]. It is well known that, due to the hierarchical nature of PES [6], conformational dynamics of native proteins is hierarchical and occurs on many different time scales corresponding to different types of molecular motions, including bond stretch and bending motions on femtoseconds, rotations of small groups (e.g. methyl) on picoseconds, side chain and backbone dihedral rotations on sub-nanoseconds to microseconds, and major domain motions up to multiple milliseconds. It is likely that water molecules play different roles in the above mentioned various type of dynamic processes. Coupling between the function and internal motions of proteins and their water environment has been intensively studied [7]–[13]. Two lines of evidences that have been presented by many experimental [14]–[20] and computational [21]–[27] reports supporting either mediating or slaving roles of water molecules are briefly summarized below.

It was demonstrated by both experimental [14] and computational studies [22] that below glass transition temperature, protein dynamics is slaved(or caged) by surrounding frozen water molecules. Protein dynamics on 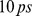 to 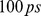 time scales was found to be closely correlated with dynamics of surrounding water hydrogen bond network and thus slaved by water molecules [23]. Water molecules’ relaxation was observed to correlate well with conformational transitions of myoglobin among statistical substates [16], which occur on time scales ranging from sub-nanoseconds to microseconds at the physiological temperature, suggesting the slaving role of water molecules on corresponding time scales.

At physiological or room temperature, certain hydration level is essential for functions of many proteins [13], [28]. Based on the analysis of crystallographic water molecules, it was proposed that water molecule “lubricate” folding of proteins through three bond centre hydrogen bonds [21]. Theoretical protein structure prediction studies [24] revealed that addition of water mediated potential in protein design facilitated search of the free energy minimum (i.e. native state), water molecules were also found to mediate native state dynamics of eglin C [26], [27]. Raman optical activity studies [15] support the role of water molecules as “lubricant of life”. From an energy landscape perspective, observations along this line were explained with the belief that water molecules facilitates protein dynamics through effectively smoothing their PES. However, direct evidence supporting this concept is lacking.

In this study, we generated collectively 5 microsecond molecular dynamics (MD) trajectories for a small globular protein barstar [29], which is synthesized by the bacterium *Bacillus amyloilyquefaciens* as an inhibitor of the ribonuclease protein barnase. By systematically analysing the time series (or evolution in conformational space) of relevant energy terms (protein self energy (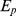), protein-water interaction energy (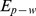) and their sum (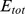)) obtained from MD trajectories, we found that while the negative correlation between 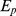 and 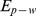 in most parts of conformational space provides possibility for PES smoothing, the dominance of 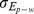 over 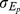 (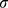 stands for standard deviation) resulting in a rougher PES on average, especially for picoseconds and longer time scales. These two aspects contribute to the end effects of water molecules on the PES of proteins, that is roughening the majority and smoothing the remaining part of the potential energy landscape. Thus, the conflicting roles of water in the protein conformational dynamics are reconciled with an energy landscape perspective. It is noted here that due to the constraint of computational resource, our analysis were limited to sub-microsecond time scale, correspond to transitions covering a few hierarchies of statistical substates. The impact of water molecules on major domain motions that occur on milli-seconds and longer time scales and folding dynamics is beyond the scope of this study.

## Results

**Figure 1:**
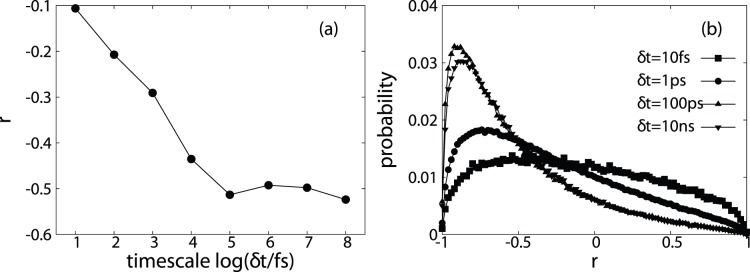
Pearson correlation coefficient *r* between *E_p_* and *E_p_*_–*w*_ for barstar. Time scale values are obtained by first reducing time scales(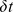) with femto-second and then taking logarithm (e.g. 1 corresponds to 10 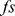, 2 corresponds to 100 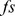, 3 corresponds to 1 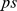, etc.) Time scales mentioned in figures hereafter are the same. (a) ensemble average of 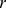 as a function of time scales. (b) Distributions of 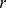 at 10 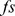 (square), 1 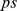 (circle), 100 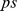 (upwards triangles) and 10 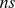 (downwards triangles).

The PES that underlies the dynamics of a protein molecule can be decomposed into two components, protein self energy (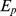) and protein-solvents interaction energy (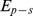). Since deciphering roles of water molecules in protein dynamics is the goal of this study, we focus our attention on 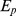 and protein-water interaction energy (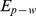). For a single protein molecule travelling in the conformational space as in a typical MD simulation or a single-molecule experiment, the PES can be represented as a time series of potential energies with its roughness represented by corresponding standard deviations.
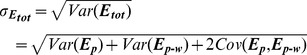
(1)where 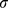 stands for standard deviation, 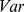 stands for variance and 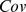 stands for covariance. A brief explanation of using standard deviation to represent PES roughness is given as follows. Unlike folding/unfolding and large scale conformational change between major conformations that occur on milliseconds and longer time scales, where one (or a few ) free energy barriers dominate, conformational transitions among a large number of hierarchical statistical substates on time scales up to microseconds involve many barriers on each specific time scale (e.g. nanoseconds) that have similar heights and are distributed over many degrees of freedoms. Therefore, we think standard deviations (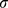) of relevant potential energy terms are a reasonable representation of PES roughness for a given time scale 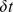. Expressing variances in eq.1 with standard deviations and the Pearson correlation coefficient 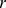 between 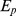 and 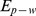, we have:



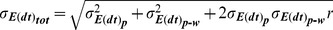
(2)

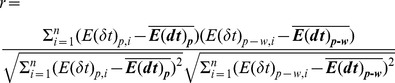
(3)where 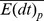 stands for the average of the consecutive 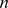 potential energy values that have 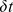 intervals in a time series 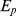. From eq.2, it is apparent that if on a given time scale 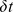, water molecules indeed smooth PES of a protein (i.e. 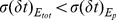), it is essential that the sum of the last two terms 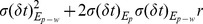 being non-positive, as standard deviations are always a non-negative number, this amounts to one of necessary conditions 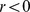. Therefore, 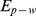 need to negatively correlate with 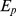 (e.g. when a protein molecule change into a higher energy configuration, water molecules compensate energetically by exerting a lower 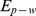). MD simulation provides great convenience in dissecting different potential energy terms on any time scales that is accessible by available computational power. To this end, we performed collectively 5 microsecond MD simulations of barstar and analysed resulting trajectories to obtain the Pearson correlation coefficients 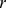 between 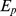 and 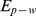. As shown in Fig. 1a, for time scales varying from 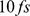 to 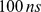 (see methods for specific procedures used to calculate energy correlation on a give time scale), 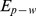 on average negatively correlate with 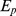, with the correlation being the minimal on the shortest time scale analysed in our study (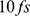), becoming stronger on longer time scales up to 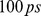 and levelling off beyond that point. However, the distribution of calculated correlation coefficient (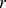) in Fig. 1b exhibit both positive and negative correlations between the two energy terms (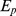 and 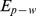), only that the negative correlation has a larger probability of occurrence.

**Figure 2:**
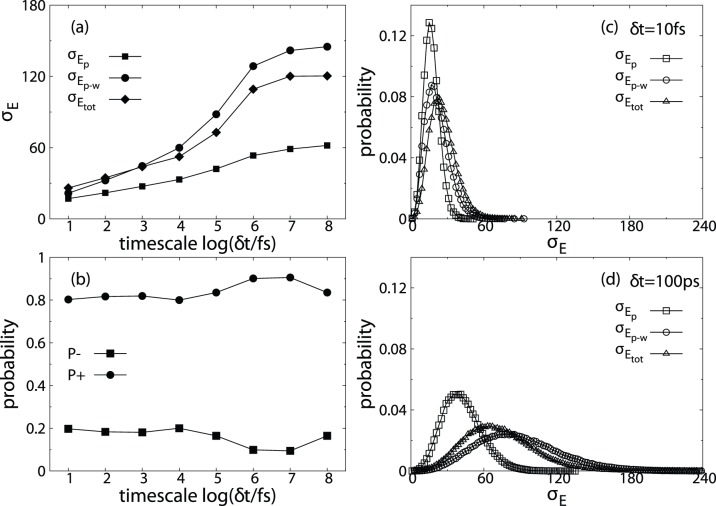
Standard deviations (σ, in the unit of *kcal*/*mol*, the same unit is used for all the following text, figures and the supporting information) for various potential energy terms of barstar. (a) 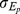 (square), 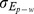
(circle) and 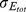 (diamond) as a function of time scales. (b) Probability of 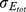 being larger than or equal to (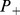, cycle) and smaller than 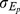 (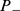, square). (c) Distributions of 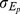 (square), 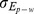 (cycle) and 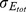 (triangle) for 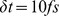. (d) Distributions of 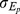 (square), 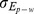 (cycle) and 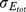 (triangle) for 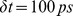.

The immediate question one would ask is that does 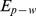, that negatively correlate with 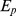, indeed smooths the PES of barstar. As mentioned above, negative correlation between 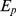 and 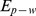 is only one of the necessary conditions. The sufficient condition is 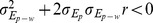 (i.e. 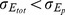). To verify this condition, we calculated the averages for the standard deviations 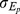, 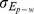 and 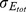, and plotted them as a function of time scales (Fig. 2a). It is apparent that for all the time scales studied, addition of protein-water interaction energies increased the roughness of the protein PES, and the roughening effects increases with increasing time scales. One interesting observation is that on short time scales (10 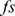 and 100 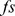), due to small negative correlation between 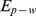 and 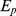, 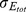 is greater than both 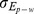 and 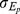. On longer time scales (10 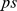 and longer), with increased negative correlation, 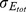 becomes smaller than 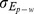 but remains greater than 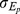. Distributions of relevant energy standard deviations for two time scales (10 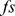 and 100 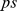, representing short and long time scales) are shown in Fig. 2c and Fig. 2d respectively. When 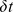 = 10 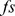, 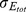 exhibits the largest spread while 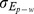 has the largest spread for 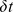 = 100 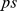. For all time scales, 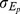 has the smallest spread. Distributions of these three energy terms for all time scales analysed are available in Fig. S1.

As ensemble averaged observables from molecular simulations has established correspondence with ensemble experimental measurements, our average PES roughness data demonstrated net roughening effects on all time scales studied, therefore unequivocally support slaving theory. Distributions of correlation coefficient 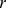 between 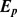 and 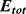 (Fig. 1b) demonstrate the complex relationship between these two different PES components. Distributions of standard deviations for the three energy terms 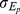, 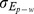 and 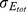 (Fig. 2c and 2d) indicate that in some region of the conformational space it is possible for 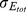 to be smaller than 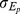. This observation suggests the possibility that water molecules do smooth corresponding part of protein PES. To validate this speculation, we compared the standard deviations calculated from each set of potential energy data (representing a specific region of PES) for 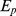 and 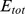, and plotted the probability that 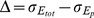 being greater or equal to (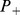, indicating roughening of PES) and smaller than 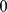 (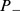, indicating smoothing of PES) as a function of time scales. The results shown in Fig. 2b demonstrate that in most parts of observed conformational space, protein-water interactions effectively roughen protein PES and play a smoothing role in the remaining part. The relative importance of the smoothing and roughening roles of water molecules shows weak dependence on time scales. The smoothing probability are larger on shorter time scales (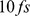 to 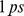) than on longer time scales (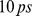 to 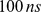).

**Figure 3:**
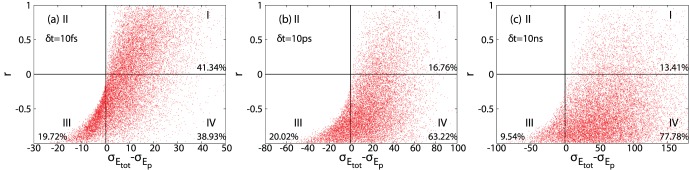
The relationship between *r* (correlation coefficient between *E_p_* and *E_p_*_–*w*_) and net effects of water molecules on local PES (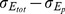) for three different time scales. a) 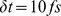, b) 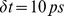 and c)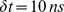. Data for all eight time scales studied are presented in Fig. S6.

As mentioned above (see also eq. 2 and 3), the net effect of water molecules on the roughness of protein PES (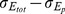) dependent on both correlation coefficient 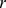 between 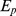 and 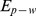 and relative magnitude of 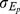 and 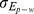. To reveal the relationship between 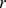 and the net effects of water molecules on barstar PES, 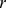 vs. 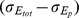 plots were generated for all the time scales that we studied and data for 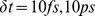 and 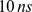 are displayed in Fig. 3a, b and c (data for other time scales are shown in Fig. S6). Each point in these plots represents a local region on a given time scale 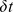 in the configurational space of barstar. In the four quadrants (noted I, II, III and IV in Fig. 3), quadrant II is always empty as it is a mathematically impossible region (from Eq. 2, if 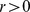, 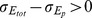), points in quadrant III corresponds to the smoothing role of water molecules, while points in quadrant I and IV correspond to roughening effects. Is it interesting to see that when 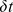 change from 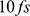 to 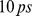, the relative weight (noted as percentage in quadrants I, III and IV) of quadrant IV increased at the expense of quadrant I, and when 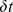 change from 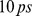 to 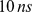, the relative weight of quadrant IV increased at the expense of both quadrant I and III, but mainly quadrant III. This observation demonstrates that on short time scale (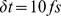), when the amplitude of 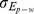 is comparable with that of 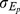 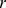 becomes the major factor of smoothing/roughening effects. On intermediate time scale (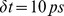), the increase in the amplitude of 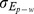 (21.6 to 59.9 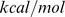, see Fig. 2a) is roughly cancelled out by the large increase in the average negative correlation (0.106 to 0.435, see Fig. 1a), thus the percentage of configurational space where water molecules smoothing protein PES remains almost the same (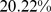 vs. 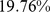). On longer time scales (e.g. 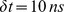), the significant increase of the average of 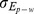 (59 to 141, see Fig. 2a) dominates the minor increase in the average negative correlation of 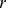 (from −0.435 to −0.497, see Fig. 1a), the percentage of configurational space where water molecules smoothing protein PES reduced significantly (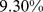 vs. 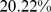).

## Discussion

On very short time scales (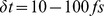), dielectric relaxation experiments revealed that protein dynamics is more or less independent of water behaviour, while slaving is mainly observed for longer time scales. This is in qualitative agreement with our data (Fig. 2a) that the difference between 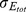 and 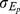 is the smallest on these time scales and monotonically increase with increasingly longer time scales. Additionally, the probability of occurrence for smoothing by water is larger on these short time scales (Fig. 2b and Fig. 3). However, the difference between 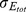 and 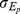 is very significant even for 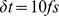 and should not be negligible in experimental dynamic measurements. This puzzle may be explained by the following fundamental physical causes that are not embodied in Eq. 2. All the bonding and bending degrees of freedom (DOFs) simultaneously contributing to the PES on femtoseconds time scales, when the limited increase of PES roughness (20 to 30 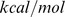) are distributed among so many DOFs (1447 bonds and 2622 angles for barstar), the net effect (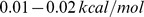 per DOF) is negligible considering large force constants of these motions (∼50 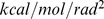 for bending and 200–500 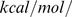 Å^2^ for bonding). However, although the total number of rotatable dihedral angles are not so small for a protein (3845 for barstar), on time scales longer than sub-nanosecond, transitions among various statistical substates mainly involve rotation of side chains (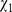) and backbone dihedral angles (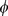 and 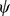) in flexible regions of protein. The total number of these dihedral angles are about three times the number residues (∼267 for barstar) and for any given region on PES, most of them are not rotatable on large scales (e.g. *trans* to *gauche*). When relatively large increase of PES roughness (∼120 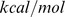) are distributed over a small number of DOFs, the net effects (∼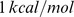 per DOF) are significant considering the small force constants (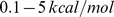) of most dihedral rotations.

Our data support both slaving and mediating roles of water molecules when different regions of PES were investigated separately. However, due to the fact that the slaving role has a larger probability of occurrence, and the fact that ensemble based experimental characterizations (e.g. photolysis analysis and dielectric relaxation measurements) are sensitive to the ensemble average of observables, thus only majority events (slaving) were seen. Conformational analysis performed on MD trajectories and PDB structures, should in principle be able to overcome such issues. However, the conformational states correspond to mediating roles of water (e.g. bridging residues of the same charge) are much easier to identify due to their relatively higher stability and structural simplicity, while a much larger number of conformational states correspond to slaving role of water molecules have lower stability and higher structural complexity. Therefore, in structural analysis of MD trajectories, the later tend to be neglected as random and featureless (which they are despite their significance in contribution to overall PES) events.

The role of water molecules is apparently even more important for the folding process of proteins as hydrophobic interactions are the most important driving force for protein folding. The critical role of individual water molecules is observed in both folding of protein molecules [30] and in triggering folding of model polymers with hydrophobic and hydrophilic monomers [31]. Both mediating [21], [24] and slaving [17] roles of water in protein folding dynamics are reported. However, due to the constraint of computational resource, we were not able to perform similar analysis for the protein folding processes.

Sampling and accuracy of force fields are the two fundamental limitations that compromise the prediction power of molecular simulation methods. In this study, we have used collectively 5 microseconds of MD simulation trajectories. Based on the success of many simulation studies we believe that our trajectories should explore a significant and representative part of the concerned protein phase space for time scales varying from multiple femtoseconds to multiple nanoseconds. The time scales investigated here cover a few hierarchies of statistical substates transitions ranging from rapid bending motion to rotation of many side chains and backbone dihedral angles. As dynamic behaviour of proteins on longer time scales (micro-to milliseconds) have been shown to be coupled with shorter time scale dynamics [32], negative correlation and dominance of protein-water interactions on these time scales likely to impact long time dynamics in some way. We hope to address this issue in the immediate future. Additionally, our analysis is mainly based upon the fluctuation of relevant energetic terms. Therefore, the absolute value of these energetic terms, which absorb a significant part of inaccuracy of the adopted molecular mechanical force fields, is not a major concern.

Two different approaches have been utilized to analyse the roles of water molecules in protein conformational dynamics. One is to monitor the behaviour of fully solvated proteins via experimental [14]–[20] or computational methodologies [21]–[27], and this is what we adopted in the current study. In this scenario, the PES (of solvated proteins) consists of two components, one is the intramolecular contribution (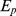) and the other is the intermolecular contribution (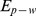). Our analyses indicate that 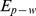 contribute more to the PES roughness than 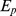. In contrast, the other approach has compared dynamical behaviour of proteins with various extents of hydration [33]–[36], where the observed difference is indisputably resulted from different extents of hydration/solvation. In studies adopting the later approach, it was found that above 250 K (below that temperature, water molecules effectively freeze up most interesting protein motions), hydration significantly enhances protein dynamics. From a PES point of view, that unequivocally leads to the conclusion that water molecules smooth protein PES. However, different conclusions from these two distinct approaches do not necessarily constitute a direct contradict. In the former approach, the relative contribution of two components of the solvated protein PES is considered, one structural ensemble (the solvated native ensemble, this is a very approximate term as different solvation conditions correspond to different ensembles) is the focus of investigation. In the later case, totally different protein PESs (that of dry proteins, fully hydrated proteins or something in between) are compared, the two extreme structural ensembles (dry and fully hydrated ensembles) are different with the extent of differences (shared and distinct conformations) being unknown. Future investigations that compare dry and solvated proteins using experimental and/or computational techniques will provide more insights.

In conclusion, by decomposing the PES 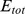 of barstar into 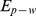 and 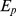, and analysing their correlations and roughness on time scales varying from femto-seconds to sub-microseconds, we found energetic evidence for both the slaving and mediating roles of water molecules. These analysis revealed that on the above mentioned time scales, in most part of the configurational space, water molecules slave protein dynamics through effectively roughening local PES and in the remaining part, water molecules may facilitate conformational dynamics by smoothing local PES. Here we carefully studied the impact of protein-water interactions on the PES of barstar from an energy landscape perspective. It is possible that other proteins with different folds may have qualitatively different behaviour. Based on the experimental reports of similar slaving behavior of many proteins [16], our conclusion is likely to be qualitatively applicable for many other globular proteins as well. It is important to note that our study focuses on the PES of fully solvated proteins, the change of dynamical behavior from dry proteins to solvated ones is not covered. As exhaustive studies of all protein folds is not achievable due to prohibitive computational cost, we hope that this study may stimulate the community’s interest to utilize available trajectories of different proteins to quantitatively answer this question, and accurately gauge the role of water molecules on protein conformational dynamics in a general sense.

## Materials and Methods

### Molecular Dynamics Simulations

All MD simulations were performed with NAMD software package [37],version 2.7 using CHARMM27 force fields. barstar (pdb code:1bta) was solvated with TIP3P water model. 100 mM 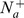 and 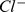 were added to neutralize net charges of our simulation systems. Bond-lengths involving hydrogen atoms were constrained using the SHAKE algorithm, and the integration time step is set to 2 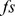. Periodic boundary conditions were used, a switch distance of 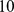
Å and a cutoff distance of 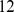 Å were used for non-bonded interactions. Particle Mesh Ewald (PME) were used to calculate the long-range electronic interactions. All systems were minimized and then heated to 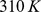 with heavy atoms restrained, water molecules were equilibrated with 200-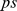 runs in NVT ensemble. After that, restraints for protein heavy atoms were released, and the whole system was equilibrated in the NPT ensemble for another 4 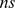. A frame with the volume value that is closest to the average volume obtained from NPT equilibration run was selected for the next production runs which were performed in the NVT ensemble at 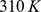. 10 500-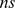 trajectories were generated. Coordinates were saved every 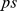 for analysis. To generate potential energy statistics on short time scales (10 and 100 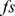). 10 100-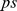 trajectories originating from snapshots taken every 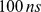 from arbitrarily selected long trajectories were generated and coordinates were saved every 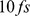.

### Energy Correlation Analysis

To measure the correlation between 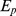 and 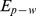 of barstar, mean value and distributions of Pearson correlation coefficients 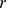 were calculated on time scales ranging from 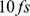 to 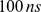. For a given time scale 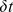, consecutive 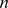 data points (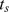, 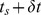, …, 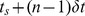) were picked from each potential energy time series(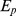 and 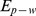) of available trajectories to calculate one Pearson correlation coefficient, 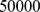 coefficients were obtained with all the 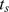 uniformly distributed in our trajectories, and were used to generate the mean and distributions. The presented data were obtained with 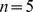, a larger 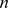
(e.g. 7,8) would mix neighbouring time scales and are therefore not used. The data for 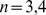 were also calculated and presented in Fig. S2. As expected, different 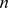 generate similar results.

### Energy Landscape Roughness Analysis

To quantitatively characterize the landscape roughness, mean and distribution of standard deviations of 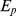, 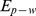 and 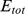 were calculated. Standard deviations of energy were calculated as its common form 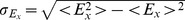, with 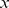 representing 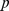, 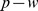 or 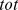, and the bracket representing average for 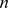 consecutive data points (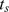, 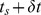, …, 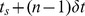) in respective energy time series. Similar to energy covariance analysis, 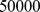 standard deviations were obtained with 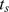 uniformly distributed in our trajectories, results from 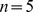 were presented in the main text and results from 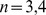 were presented in the supplementary material (Fig. S3, S4 and S5).

## Supporting Information

Figure S1
**Distributions of energy standard deviations 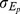 (square), 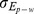 (circle) and 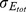 (triangle) for barstar on various time scales.** (a) 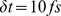, (b) 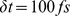, (c) 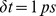, (d) 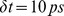, (e) 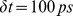, (f) 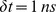, (g) 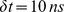 and (h) 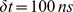.(EPS)

Figure S2
**Average Pearson correlation coefficient 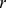 between 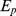 and 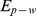 calculated with various 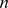 (see Eq. 3) as a function of time scale for barstar.**
(EPS)

Figure S3Distributions of protein self energy standard deviations 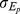 calculated with 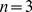 (red), 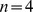 (green) and 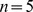 (blue) for (a) 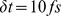, (b) 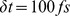, (c) 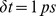, (d) 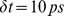, (e) 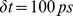, (f) 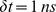, (g) 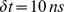 and (h) 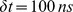.(EPS)

Figure S4Distributions of protein-water interaction energy standard deviations 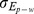 calculated with 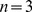 (red), 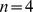 (green) and 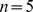 for (a) 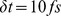, (b) 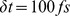, (c) 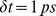, (d) 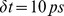, (e) 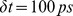, (f) 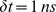, (g) 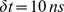 and (h) 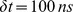.(EPS)

Figure S5Distributions of total energy standard deviations 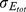 calculated with 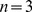 (red), 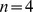 (green) and 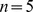 (blue) for (a) 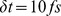, (b) 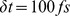, (c) 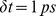, (d) 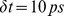, (e) 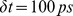, (f) 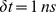, (g) 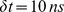 and (h) 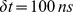.(EPS)

Figure S6
**The relationship between 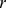 (correlation coefficient between 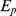 and 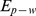) and net effects of water molecules on local PES (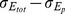) for eight different time scales.** (a) 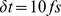, (b) 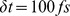, (c) 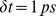, (d) 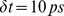, (e) 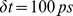, (f) 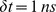, (g) 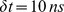 and (h) 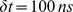.(EPS)
